# Criteria-Based Audit of Quality of Care to Women with Severe Pre-Eclampsia and Eclampsia in a Referral Hospital in Accra, Ghana

**DOI:** 10.1371/journal.pone.0125749

**Published:** 2015-04-29

**Authors:** Joyce L. Browne, Sabine W. van Nievelt, Emmanuel K. Srofenyoh, Diederick E. Grobbee, Kerstin Klipstein-Grobusch

**Affiliations:** 1 Julius Global Health, Julius Centrum for Health Sciences and Primary Care, University Medical Center Utrecht, Utrecht, the Netherlands; 2 Ridge Regional Hospital, Ghana Health Service, Greater Accra Region, Accra, Ghana; 3 Division of Epidemiology & Biostatistics, School of Public Health, Faculty of Health Sciences, University of the Witwatersrand, Johannesburg, South Africa; St Francis Hospital, UNITED STATES

## Abstract

**Objectives:**

Severe pre-eclampsia and eclampsia are one of the major causes of maternal mortality globally. Reducing maternal morbidity and mortality demands optimizing quality of care. Criteria-based audits are a tool to define, assess and improve quality of care. The aim of this study was to determine applicability of a criteria-based audit to assess quality of care delivered to women with severe hypertensive disorders in pregnancy, and to assess adherence to protocols and quality of care provided at a regional hospital in Accra, Ghana.

**Methods:**

Checklists for management of severe preeclampsia, hypertensive emergency and eclampsia were developed in an audit cycle based on nine existing key clinical care protocols. Fifty cases were audited to assess quality of care, defined as adherence to protocols. Analysis was stratified for complicated cases, defined as (imminent) eclampsia, perinatal mortality and/or one or more WHO maternal near miss C-criteria.

**Results:**

Mean adherence to the nine protocols ranged from 15–85%. Protocols for ‘plan for delivery’ and ‘magnesium sulphate administration’ were best adhered to (85%), followed by adherence to protocols for ‘eclampsia’ (64%), ‘severe pre-eclampsia at admission’ (60%), ‘severe pre-eclampsia ward follow-up’ (53%) and ‘hypertensive emergency’ (53%). Protocols for monitoring were least adhered to (15%). No difference was observed for severe disease. Increased awareness, protocol-based training of staff, and clear task assignment were identified as contributors to better adherence.

**Conclusion:**

A criteria-based audit is an effective tool to determine quality of care, identify gaps in standard of care, and allow for monitoring and evaluation in a health facility, ultimately resulting in improved quality of care provided and reduced maternal morbidity and mortality. In our audit, good adherence was observed for plan for delivery and treatment with magnesium sulphate. Substandard adherence to a number of protocols was identified, and points towards opportunities for targeted improvement strategies.

## Introduction

Reducing the maternal mortality rate (MMR) by three quarters is one of the Millennium Development Goals (MDGs). Since 1990, significant successes have been made [1]: the average global MMR reduced from 400 per 100.000 live births in 1990 to 210 per 100.000 live births in 2013 [2]. However, progress is insufficient and more needs to be done to reach this MDG, warranting further efforts and continued focus on maternal health post 2015 [3]. In low- and middle-income countries (LMICs) the main causes of maternal mortality are hemorrhage, sepsis, unsafe abortion and hypertensive disorders in pregnancy [4,5]. The most life-threatening complications of hypertensive disorders, pre-eclampsia, eclampsia and HELLP syndrome (hemolysis, elevated liver enzymes, and low platelets), account globally for 10–15% of maternal mortality, with over 50.000 annual maternal deaths. Almost all occur in LMICs, with concurrent high perinatal mortality and morbidity [5,6]. Therefore, optimization of the management of pre-eclampsia and eclampsia will protect women and newborns from complications and death.

**Fig 1:**
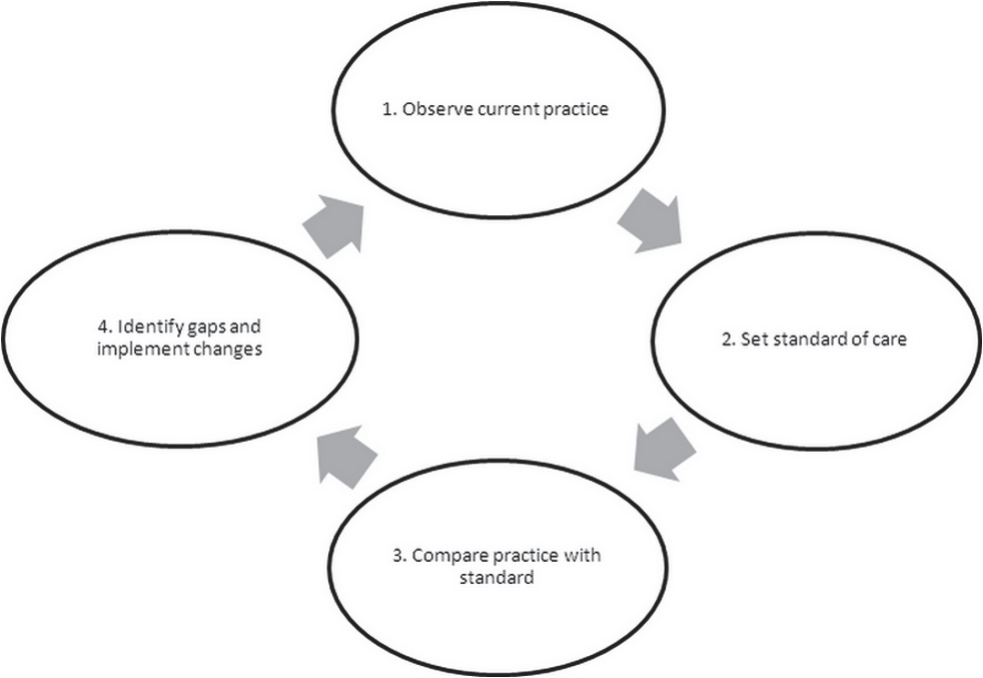
The audit cycle (adapted from Crombie et al 1993) [21].

A criteria-based audit (CBA) is, in both high-income countries and LMICs, a useful method to define and improve quality of care regarding management of severe pre-eclampsia and eclampsia [7–20]. An audit is a cyclical process of defining standards of care, collecting data of adherence to standards, identifying areas for improvement, implementing necessary changes and back to defining new standards as indicated in Fig 1 [21]. In a CBA, health staff agrees on a set of appropriate and locally adapted management criteria, and subsequently an independent reviewer screens the records of patients to determine whether care provided meets the agreed criteria [22]. Finally, results are discussed and changes implemented in the system of current practice [22].

A CBA of severe maternal complications and near-miss cases allows a complete assessment of quality of care (QoC) in life-threatening conditions and explores the similarities, differences and the relationship between women who died and those who survived [11,23,24]. As the survival group experienced a significant and similar scale of complications as those who die, survival could be related to the QoC received and optimal care components identified [23,25].

Maternal mortality rate in Ghana in 2010 was 350 per 100.000 live births; this is 100–200 times higher than the MMR in Europe and North America [4,5]. Since 2007 the Ridge Regional Hospital in the Accra Metropolis in Ghana, has been one of the expertise centres in the cooperation between the Ghana Health Service (GHS) and Kybele, a non-governmental organization based in the United States [26]. The collaboration involves joint efforts to identify and address bottlenecks within the system to improve maternal and neonatal outcomes. One of the outputs of this collaboration is the development of clinical guidelines, protocols and standard operating procedures for the management of major obstetric complications.

The aim of this study was twofold, first to determine applicability of a CBA to assess QoC delivered to women with severe hypertensive disorders in pregnancy, and secondly to assess adherence to protocols and QoC provided at a regional hospital in Accra, Ghana.

## Subjects and Methods

### Study design

A mixed-methods approach was applied to accomplish the audit cycle (Fig 1). This included observation of current practice by group interviews and shadowing sessions, creation of a set of criteria based on the existing hospital guidelines from 2007, and a cross-sectional study on adherence to the guidelines by auditing cases with severe hypertensive disorders in pregnancy.

### Development of CBA

To observe current practice a house officer was shadowed during a shift on the obstetric emergency room and four midwives of the obstetric ward participated in a semi-structured group interview. In this focus group the management of severe pre-eclampsia, hypertensive emergency, and eclampsia; the current clinical situation, usefulness of protocols, opinions about protocols and suggestions for changes in current practice on the ward were discussed. Observations of the shadowing session and focus group were discussed within the study group, which included the Head of the Obstetrics & Gynaecology Department.

The local guideline of Ridge Regional Hospital was compared to two international guidelines of the World Health Organization (WHO) [27] and the International Society of the Study of Hypertension in Pregnancy (ISSHP) [28], and two national guidelines of Safe Motherhood [29] and the Ghana Ministry of Health [30]. Differences were discussed with the research group and clinical staff, and thereafter consensus criteria for standards of care were developed. The guideline was divided in nine key protocols with sub-protocols, each transformed in a checklist of criteria (Table 1). The checklists are available as a supplement to this article (S1 Fig).

**Table 1:** Protocols used as standard of care with number of criteria per protocol, Ridge Regional Hospital, Accra, Ghana, 2013.

Protocol	Number of criteria (in sub-protocols)
Severe pre-eclampsia at admission	History: 8 criteria
	Physical examination: 12 criteria
	Investigations: 9 criteria
	Non-pharmacological treatment: 2 criteria
	Pharmacological treatment: 9 criteria
Severe pre-eclampsia at follow-up	Monitoring: 6 criteria
Plan for delivery	GA 26–31 weeks: 10 criteria
	GA 32–34 weeks: 6 criteria
	GA > 34 weeks: 1 criterion
Hypertensive emergency	Preparation: 2 criteria
	Acute examination: 6 criteria
	Acute treatment: 11 criteria
Eclampsia	Non-pharmacological treatment during fit: 11 criteria
	Non-pharmacological treatment after fit: 12 criteria
	Pharmacological treatment: 5 criteria
Magnesium sulphate protocol	6 criteria
Hydralazine protocol	10 criteria
Emergency monitoring protocol	5 criteria
Stable monitoring protocol	3 criteria

### Application of CBA

Audits took place in May and June 2013 on week days. Cases recruited for the CBA were women diagnosed with severe pre-eclampsia, hypertensive emergency or eclampsia during pregnancy, as they represent severe hypertensive disorders in pregnancy [27,28]. Inclusion criteria were presence of at least one of the following: proteinuria of ≥2+ on an urine dipstick, systolic blood pressure of ≥160 mmHg, diastolic blood pressure of ≥110 mmHg, or eclamptic seizures. Participants were classified as “complicated” or “uncomplicated pre-eclampsia”. Complicated cases of pre-eclampsia were defined as those with imminence or presence of eclampsia, perinatal mortality and/or one or more WHO criteria of a life-threatening condition during pregnancy, delivery or 48 hours after delivery, based on the WHO criteria to define near-miss cases and locally adapted by adding imminent eclampsia as a major cause of maternal mortality and near misses [12,25]. All cases of severe hypertensive disorders in pregnancy presenting in this period were included, as the severe morbidity of the patients required admission for more than two days at the ward, ensuring data collection before patients were discharged.

CBA data collection comprised of two parts: first, an interview to collect maternal socio-demographic, socio-economic and clinical information with a standardized questionnaire and second by screening patient records for evidence of management activities per relevant checklists. Analysis was performed for adherence of health care professionals to the nine protocols, defined as both conducted and reported in a patient record, for the total population and by complication group.

### Outcomes

Applicability was defined as the degree to which the CBA could be used in this setting. Specifically, we assessed whether the audit cycle could be performed. According to the criteria set by the International Federation of Gynaecology and Obstetrics (FIGO) and the WHO an audit cycle can be performed when (1) a standard of good practice is available, (2) health care personnel feels able to openly discuss case management without feeling threatened and is willing to envisage the application of revised protocols for care and (3) facility registers are available to identify relevant cases [31,32]. We also assessed whether the routine data recorded in the care of patients could be used, and whether the underlying assumption that “if it is not recorded to be performed, it did not happen” was appropriate. For four specific criteria (blood pressure monitoring and registration, cardiotocography at admission, ultrasound at admission and input/output monitoring and charting) the more subtle dimensions of adherence was assessed and the difference between “was there a plan [an order] made?” and “was this plan [the order] executed?” observed based on absence or presence of documentation in the patient folder by physicians and nurses. These four criteria were selected based on the available trace in the records when they would have been completed. Four points were assigned to cases in which it was both planned and the execution registered, fewer if one of the two or both were missing.

### Statistical methods

Data was entered into a database using EpiDataEntry version 3.1 [33], cleaned and checked for missing and not applicable data. “Not applicable” data was defined as criteria that were not relevant or applicable in the management of the participant. Demographic, economic and maternal characteristics stratified by complication group were analyzed using Student’s t-test for continuous normal distributed variables, Mann-Whitney U test for continuous abnormal distributed variables and Pearson’s Chi-square test for categorical variables. Descriptive analysis in percentages was performed for adherence to particular items of the protocols, of which mean adherence to the full protocols were extracted. Differences in percentage of adherence to particular items of protocols by complication group and by socio-demographic status (parity, employment, maternal education, marital status) were analyzed using Pearson’s Chi-square test. Level of statistical significance was set at p<0.05. Analysis was performed using SPSS version 20 [34].

### Ethical clearance

Ethical approval for this study was obtained from the Ghana Health Services Ethical Review Committee [GHS-ERC 07/9/11]. Written informed consent was obtained from participants prior to inclusion.

## Results

### Study population

The management of 50 patients with severe pre-eclampsia, hypertensive emergency or eclampsia was audited. Of the 50 women, 13 out of 50 (26%) had a hypertensive emergency, and 11 out of 50 (22%) eclampsia. After admission 24 of 50 (48%) women delivered at the same day and 26 (52%) were admitted for one or more days until delivery. Table 2 illustrates the baseline characteristics. Participants were on average 29.9 years (standard deviation (SD) 6.2) with a mean gestational age at day of admission of 35.6 weeks (SD 4.09); 56% were multiparous, on the average with their second pregnancy (mean 2.14, SD 1.15).

**Table 2:** Baseline characteristics of study population and incidence of complications, stratified by uncomplicated and complicated severe pre-eclampsia (n = 50), Ridge Regional Hospital, Accra, Ghana, 2013.

	Total population (n = 50)		Uncomplicated* severe pre-eclampsia (n = 23)		Complicated* severe pre-eclampsia (n = 27)		*P*—value**
Demographic and obstetric variables	Mean	SD	Mean	SD	Mean	SD	
Age (n = 50)	29.9	6.2	30.4	5	29.5	7.0	0.622^a^
Systolic blood pressure (mmHg, n = 46)	173	28	172	21	174	34	0.810^a^
Diastolic blood pressure (mmHg, n = 46)	111	18	112	12	110	23	0.991^b^
Gestational age in weeks (n = 48)	35.6	4.1	36.4	4.5	34.9	3.7	0.190^b^
Multiparity (n = 28)	2.1	1.2	2.2	0.9	2.1	1.4	0.630^b^
	**n**	**%**	**n**	**%**	**n**	**%**	
Parity (n = 50)							0.945^c^
*Nullipara*	22	44	10	43.5	12	44.4	
*Multipara*	28	56	13	56.5	15	55.6	
Gestational age (n = 48)							0.488^c^
*>34 weeks*	34	68	18	78.3	16	64	
*32–34 weeks*	4	8	1	4.3	3	12	
*26–31 weeks*	10	20	4	17.4	6	24	
Mode of delivery (n = 50)							**0.075** ^**c**^
*SVD*	4	8	4	17.4	0	0	
*Emergency CS*	44	88	18	78.3	26	96.3	
*Elective CS*	2	4	1	4.3	1	3.7	
Marital status (39)							0.268^c^
*Single*	5	19	2	11.8	3	13.6	
*Married*	31	62	15	88.2	16	72.7	
*Living with partner*	3	6	0	0	3	13.6	
Religion (n = 39)							0.376^c^
*Christian*	32	82.1	15	88.2	17	77.3	
*Muslim*	7	17.9	2	11.8	5	22.7	
Highest educational level (n = 39)							0.247^c^
*No education*	7	17.9	2	11.8	5	22.7	
*Primary school*	10	25.6	4	23.5	6	27.3	
*Junior High School*	12	30.8	6	35.3	6	27.3	
*Senior High School*	3	7.7	0	0	3	13.6	
*Professional/vocational school*	7	17.9	5	29.4	2	9.1	
Employment (n = 39)							0.582^c^
*Yes*	33	84.6	15	88.2	18	81.8	
*No*	6	15.4	2	11.8	4	18.2	
**Complications**	**n**	**%**	**n**	**%**	**n**	**%**	
Eclampsia	11	22.0	NA		11	40.7	
Imminent eclampsia	15	30.0	NA		15	55.6	
Perinatal mortality	2	4.0	NA		2	7.4	
One or more WHO criteria of life-threatening conditions	12	24	NA		12	44.4	
*1 WHO criterion*	8	16	NA		8	29.6	
*2 WHO criteria*	4	8	NA		4	14.8	

*Complicated severe preeclampsia includes: ‘imminent of eclampsia’, ‘eclampsia’, ‘perinatal mortality’ and ‘one or more WHO criteria of life-threatening conditions’

** Significant at P<0.05

SD = standard deviation, SVD = spontaneous vaginal delivery, CS = caesarian section

^a^ = Student’s t-test

^b^ = Mann-Whitney U test

^c^ = Pearson’s Chi-square test

### Applicability

**Fig 2:**
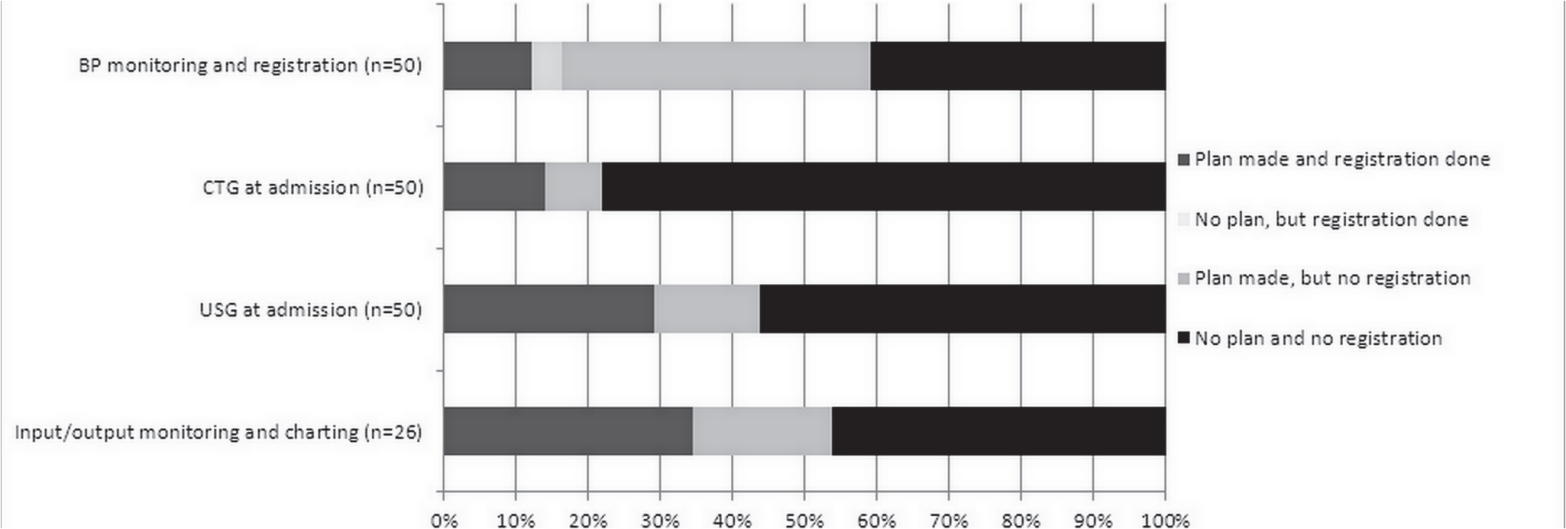
Level of adherence to four specific criteria of the protocol of severe pre-eclampsia (n = 50), Ridge Regional Hospital, Accra, Ghana, 2013.

We were able to apply the audit cycle in this study as all factors were fulfilled. Fig 2 illustrates the level of adherence to four specific criteria. In these four criteria, in less than 40% the plan and execution were recorded. In a number of cases a plan was made, but execution was not recorded (8–42%), and rarely did execution take place without a plan (4%).

### General adherence to protocols

**Fig 3:**
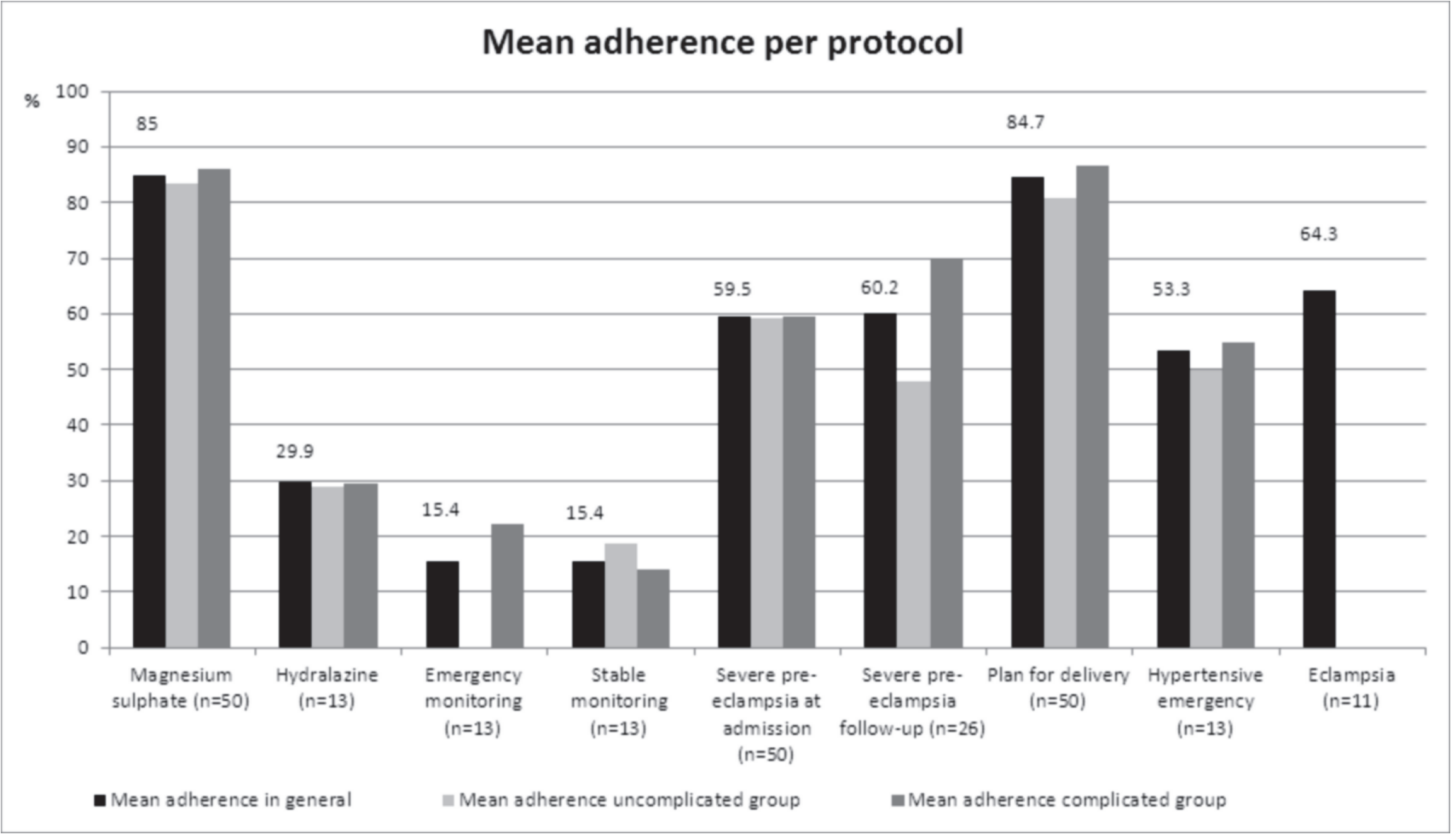
Mean adherence per protocol for total study sample and stratified by uncomplicated and complicated severe pre-eclampsia (n = 50), Ridge Regional Hospital, Accra, Ghana, 2013.

Fig 3 shows mean accumulative adherence to the nine protocols for the total study population. Mean adherence to the protocols for severe pre-eclampsia at admission and at follow-up was 60% (range 0–98% and 35–96%, respectively). Protocol for plan of delivery (differentiated by gestational age at admission of 26–31 weeks, 32–34 weeks and >34 weeks) had a mean adherence of 84%, 85% and 94%, respectively. The eclampsia protocol showed a mean adherence of 64% (range 0–100%), with four women eventually experiencing one or more eclamptic convulsion on the ward. For none of these eclamptic convulsions management during the fit(s) was fully described in the patient folders. As a result, a mean adherence to protocol of non-pharmacological treatment *during* a fit of 26% was seen. For the protocols of non-pharmacological and pharmacological treatment *after* a fit a mean adherence of 68% and 74% was observed.

In the protocol for magnesium sulphate (MgSO_4_) the item ‘continue maintenance dose till 24 hours postpartum’ had the lowest adherence to with 45%. The other five items of the MgSO_4_ protocol scored between 86% and 98% adherence. Of the hydralazine protocol, drug management steps were closely adhered to (92% adherence in the first step to administer 10mg IV; 83% adherence in step 14 of a total maximum doses of 40mg) as well as requesting for supervision: house officer and consultant were requested in 67% of cases. However, all other items in MgSO_4_ protocol had an adherence of 50 percent or lower. All monitoring protocols had low adherence scores. Analyses between socio-demographic characteristics of patients and adherence to protocols showed higher adherence for the protocols magnesium sulphate and severe pre-eclampsia at admission in multipara patients, however no relation of socio-demographic factors with any of the other protocols was observed (data not shown).

### Adherence to protocols in complicated vs. uncomplicated group

Table 2 presents incidence of each complication included in our definition of ‘complicated severe pre-eclampsia’. Among the 54% of participants who developed one or more complications, imminent eclampsia occurred most, followed by presence of at least one WHO life-threatening criteria, and eclampsia. Two cases of perinatal mortality occurred. Of all maternal characteristics it is only in the mode of delivery that a trend towards significant difference between women without and women with complications could be observed. Emergency caesarean section was conducted in 78.3% of participants in the uncomplicated group versus 96.3% of participants in the complicated group (p = 0.075). All four spontaneous vaginal deliveries occurred in women without any complications and 61% of the caesarean sections occurred in women with complications.

Fig 3 illustrates the comparison of mean adherence to eight protocols between the group of women with uncomplicated severe pre-eclampsia and the group with complicated severe pre-eclampsia. The protocol of “eclampsia” only applied to the complications strata. No significant difference was found between the uncomplicated and complicated group in adherence to the protocols of severe pre-eclampsia at admission (59% vs. 60%, p = 0.922), plan for delivery (81% vs. 87%, p = 0.904), hypertensive emergency (50% vs 55%, p = 0.620), magnesium sulphate (83% vs. 86%, p = 0.922), hydralazine (29% vs. 29%, p = 0.561), emergency monitoring (0% vs. 22%, p = 0.132) and stable monitoring (19% vs. 14%, p = 0.925). A trend towards significant difference was observed in adherence to the protocol of severe pre-eclampsia at follow-up (48% vs. 70%, p = 0.057). No significant difference was observed between both groups in adherence in sub-protocols, with the exception for history taking of women with severe pre-eclampsia at admission (80% vs. 64% adherence, p = 0.035).

## Discussion

As pre-eclampsia and related complications are the second major cause of the MMR in Ghana and many other LMICs, the government and many hospitals created protocols to optimize quality of care to women presenting with hypertensive disorders. In Ridge Regional Hospital, the CBA could be applied and 50 cases were audited. These showed a remarkably good adherence to some protocols (e.g. magnesium sulphate and plan for delivery) yet several gaps in adherence to others, suggesting sub-standard care. The congruence between planning, execution and recording of the execution of orders suggest that the “if it is not recorded, it did not happen” approach in these settings is appropriate and can be used in subsequent CBAs.

Despite the existence of guidelines and protocols, a gap between recommended care and clinical practice often exist [33, 34]. A systematic review of the quality of health delivered to adults in the United States showed that patients received about 54.9% of recommended care [35]. Factors that influence adherence to clinical guidelines and protocols include the implementation strategies, characteristics of guidelines, professionals involved, characteristics of the patients and the environment [36]. In this study, the group interviews with staff of the obstetrics ward suggested possible explanations for difference in adherence. Protocols that received more attention in the hospital (e.g. visible reminders on the wall, recent training of nurses and midwives) were adhered to more closely. A precise and clear allocation of tasks was found to be a factor promoting adherence: the magnesium sulphate treatment protocol assigns responsibility to two staff members at a given time, whereas other protocols generally included more staff members. Other factors present at Ridge Regional Hospital were occasional unavailability of emergency medication such as hydralazine, the frequent breakdown of monitoring equipment and staff shortages. Further, a strong sense of ownership can be achieved by including opinions of local staff in the agreed criteria [21], which was part of the quality improvement program at Ridge Regional Hospital with Kybele [26]. This initiative was followed by a 35% reduction of maternal mortality and 40% reduction in institutional stillbirths since the start of the program in 2007 [35].

For inclusion eligibility of this study, we applied a modified version of WHO’s severe pregnancy complication and near-miss definitions developed by Nelissen et al., [12] for low-resources settings. This near miss-definition includes (imminent) eclampsia as a criteria. To assess the impact of this classification choice, we evaluated the impact of applying the WHO ‘C-criteria’ linked to organ system dysfunction [25]. This resulted in 24% of our study population classified as near-miss, compared to the 54% observed with Nelissen’s classification, hinting towards a possible underestimation of near-miss cases with the WHO definition. This is in agreement with the CBA applied by Van den Akker et al. [36] in Malawi that showed an overall near-miss prevalence of 22% when WHOs ‘C-criteria’ were used for definition of cases, compared to 88% percent of the study population which could be classified as near-miss based on disease-specific criteria (‘A-Criteria’), which includes eclampsia. However, the percentage of ‘A-criteria’ classified (pre-) eclampsia patients (23% of their study population), that met the ‘C-criteria’ near-miss classification, could not be disaggregated in this study. Likewise, a systematic review of 82 studies by Tunçalp et al., [37] suggest a lower reporting of near-miss using organ dysfunction criteria, compared to disease-specific criteria.

A CBA is considered not only a valuable tool to determine gaps in standard of care [19,20], but also for monitoring and evaluation (M&E) as it establishes the baseline of care, and facilitates monitoring of care, and post-intervention assessment to determine improvement in quality of care during a certain period of time [14,18–20]. Especially for routine monitoring purposes, a reduction of the number of criteria in our checklist in a process locally steered by the midwives, nurses and gynaecologists, is recommended. This corresponds to observations in other CBA studies in Sub-Saharan countries that a smaller number of criteria increases the feasibility to implement CBAs in clinical practice [10,12,14,16,17,19,20].

## Conclusion

A CBA for quality of care provided to women with severe hypertensive disorders in pregnancy is a process based on local guidelines and contexts. Substandard adherence to a number of protocols can be identified and point towards opportunities for targeted improvement strategies. The CBA is an effective tool to determine quality of care, identify gaps in standard of care, and allow for monitoring and evaluation in a health facility, ultimately resulting in improved quality of care provided and reduced maternal morbidity and mortality.

## Supporting Information

S1 Fig Checklists to perform the audit categorized by protocol. (ZIP)
