# Supplementary figures and images for: Criteria-Based Audit of Quality of Care to Women with Severe Pre-Eclampsia and Eclampsia in a Referral Hospital in Accra, Ghana
Source: PLoS One. 2015 Apr 29;10(4):e0125749. doi: 10.1371/journal.pone.0125749 (PMC4414498; doi:10.1371/journal.pone.0125749)

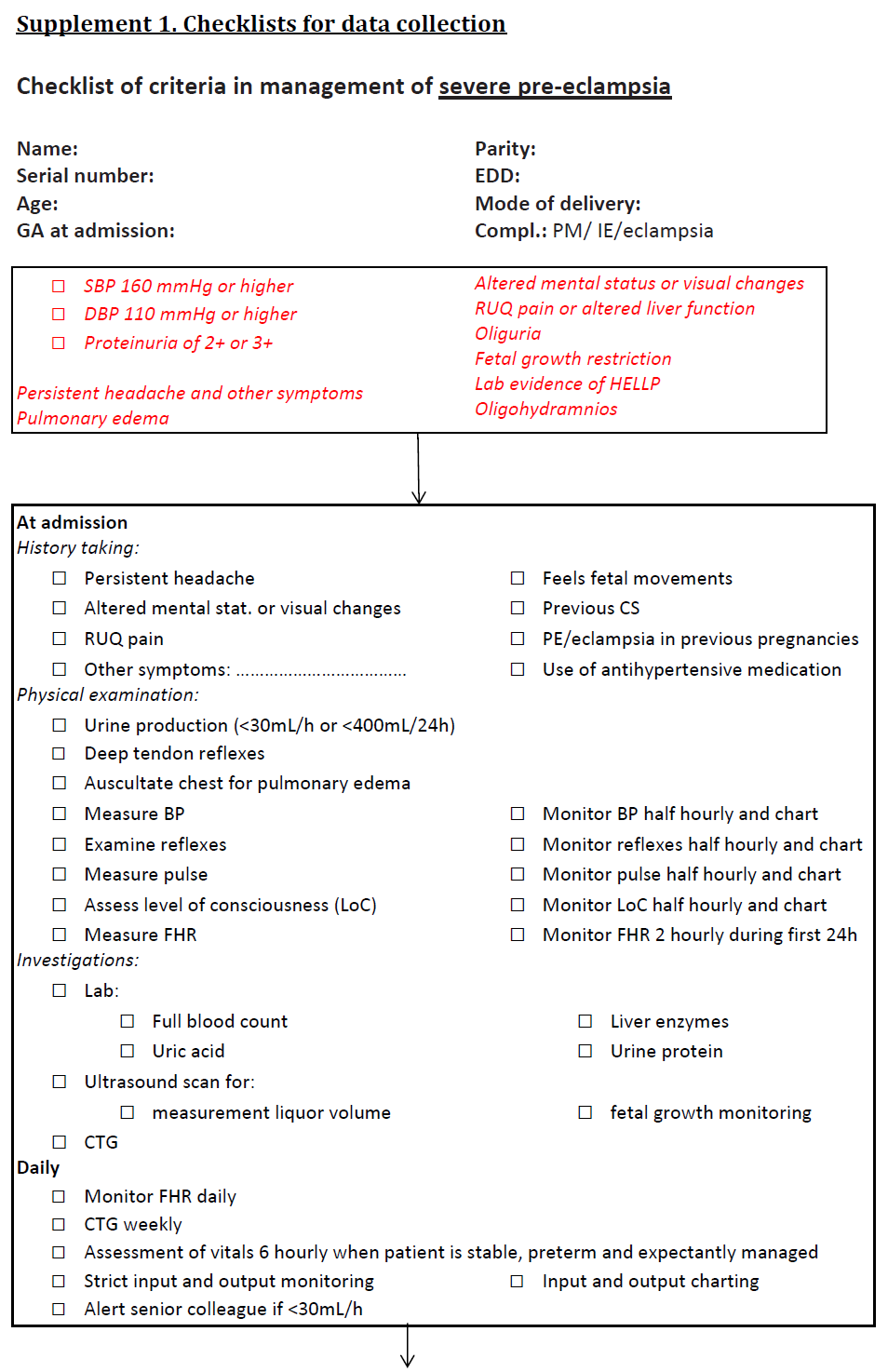

Supplement: S1 Fig — (ZIP) [file pone.0125749.s001.zip › Supplement 1-1.tif]

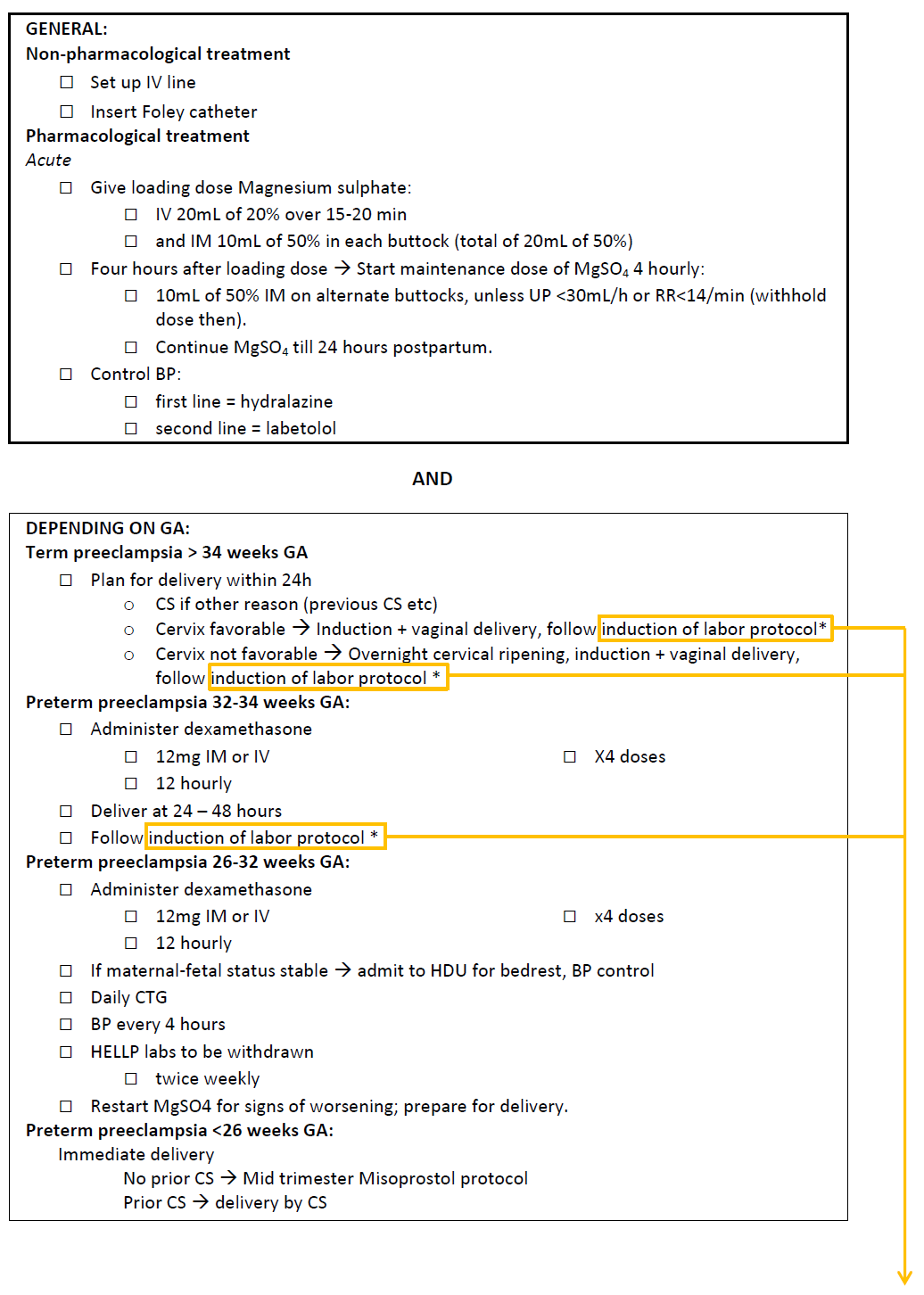

Supplement: S1 Fig — (ZIP) [file pone.0125749.s001.zip › Supplement 1-2.tif]

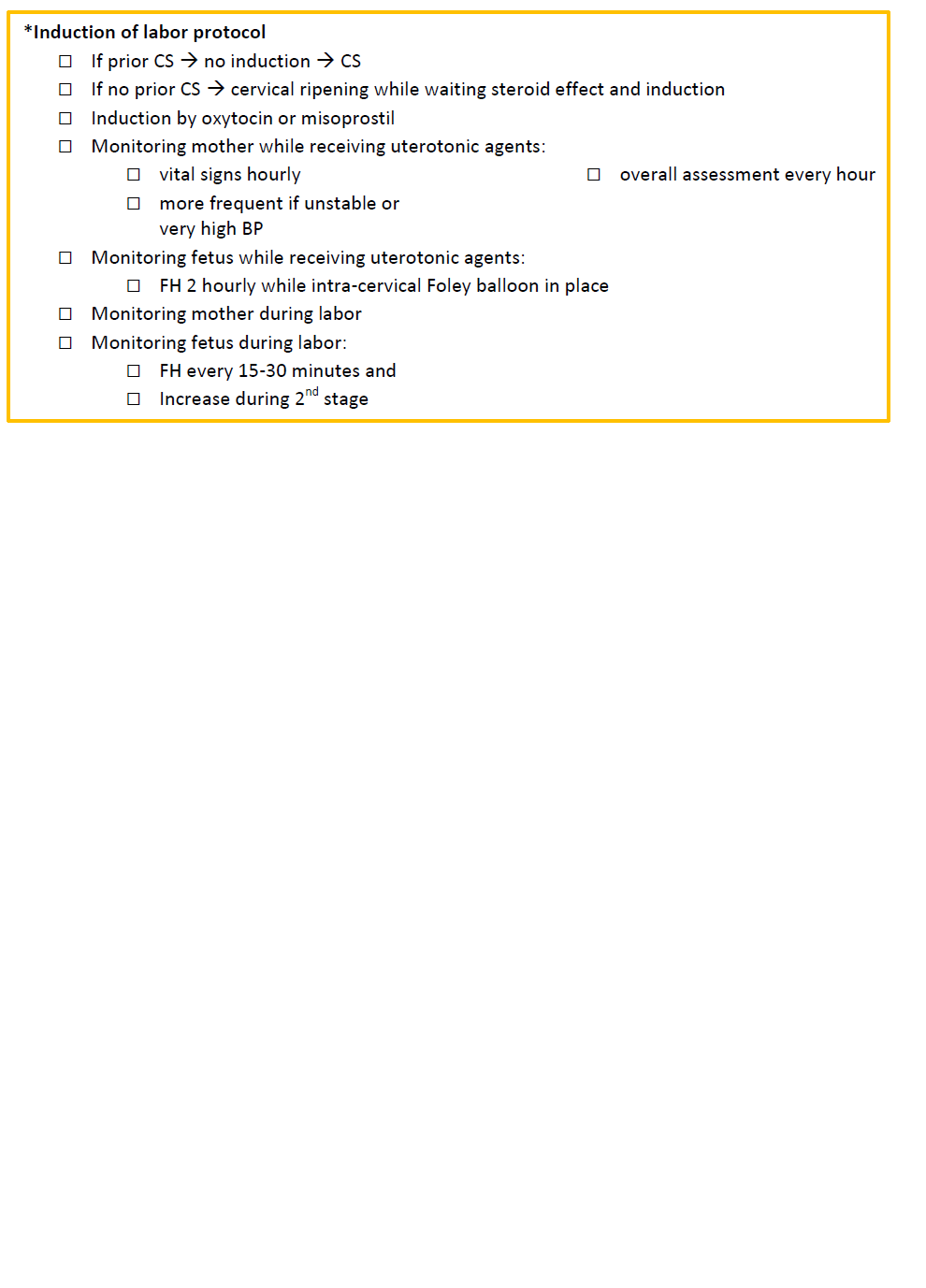

Supplement: S1 Fig — (ZIP) [file pone.0125749.s001.zip › Supplement 1-3.tif]

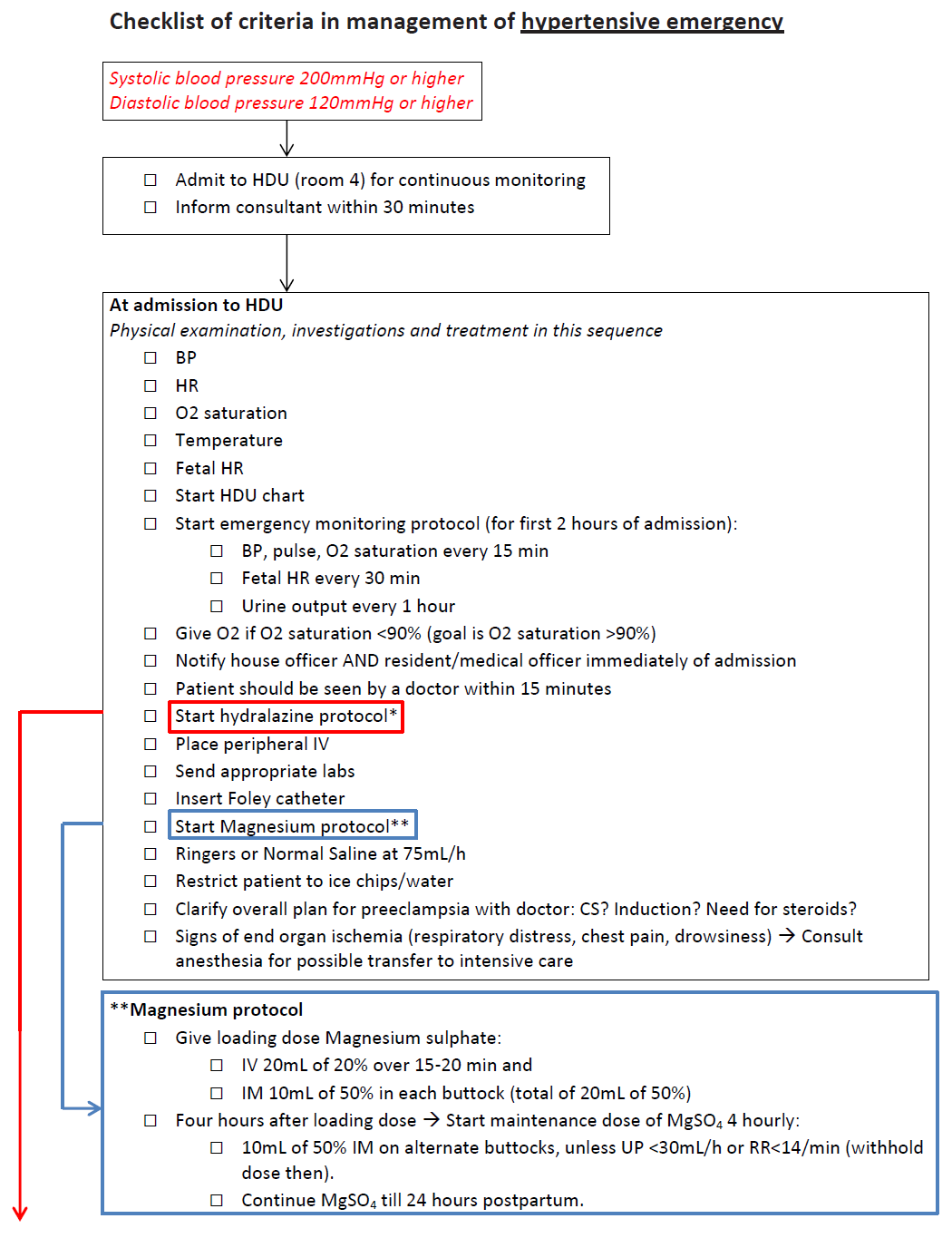

Supplement: S1 Fig — (ZIP) [file pone.0125749.s001.zip › Supplement 1-4.tif]

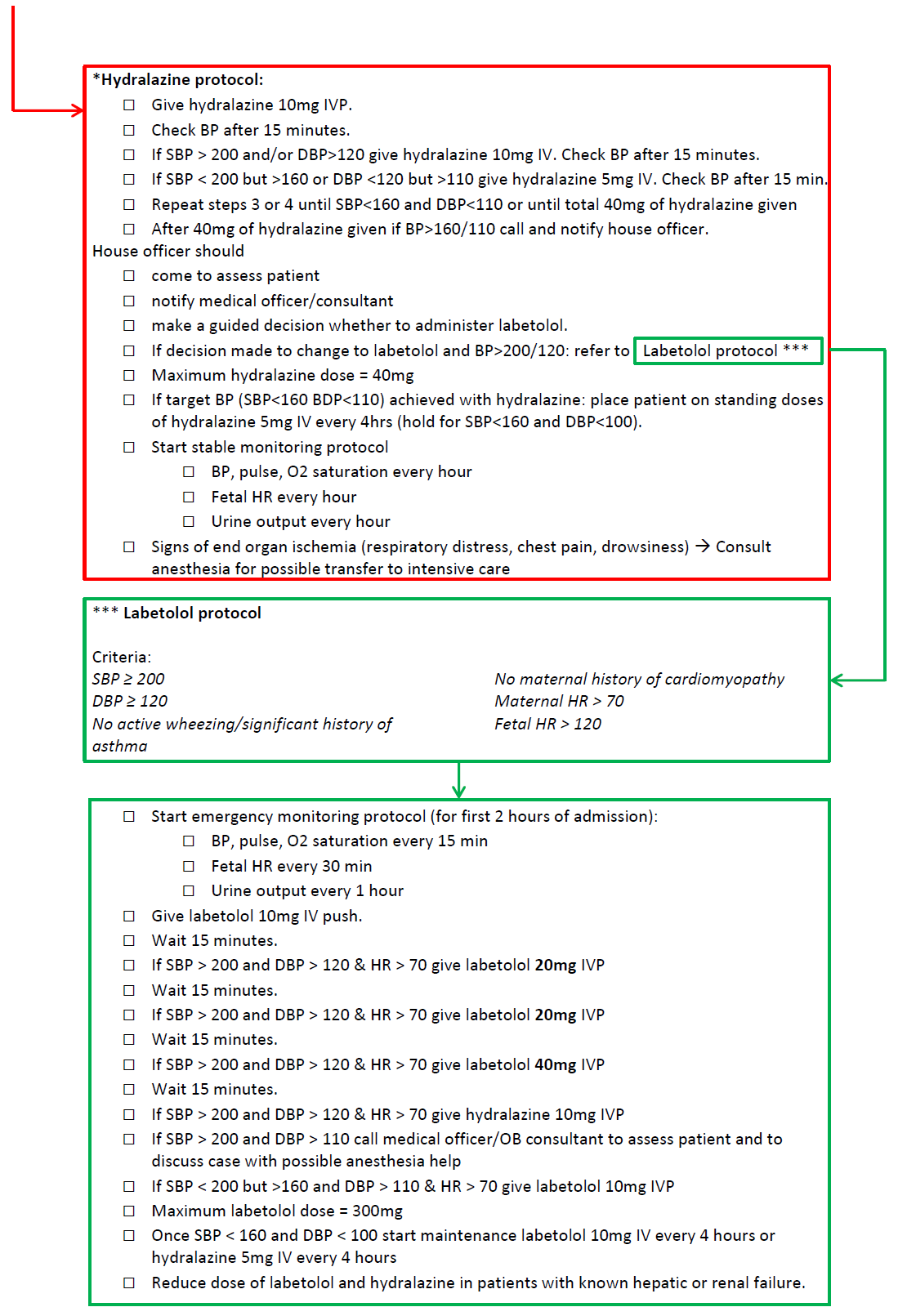

Supplement: S1 Fig — (ZIP) [file pone.0125749.s001.zip › Supplement 1-5.tif]

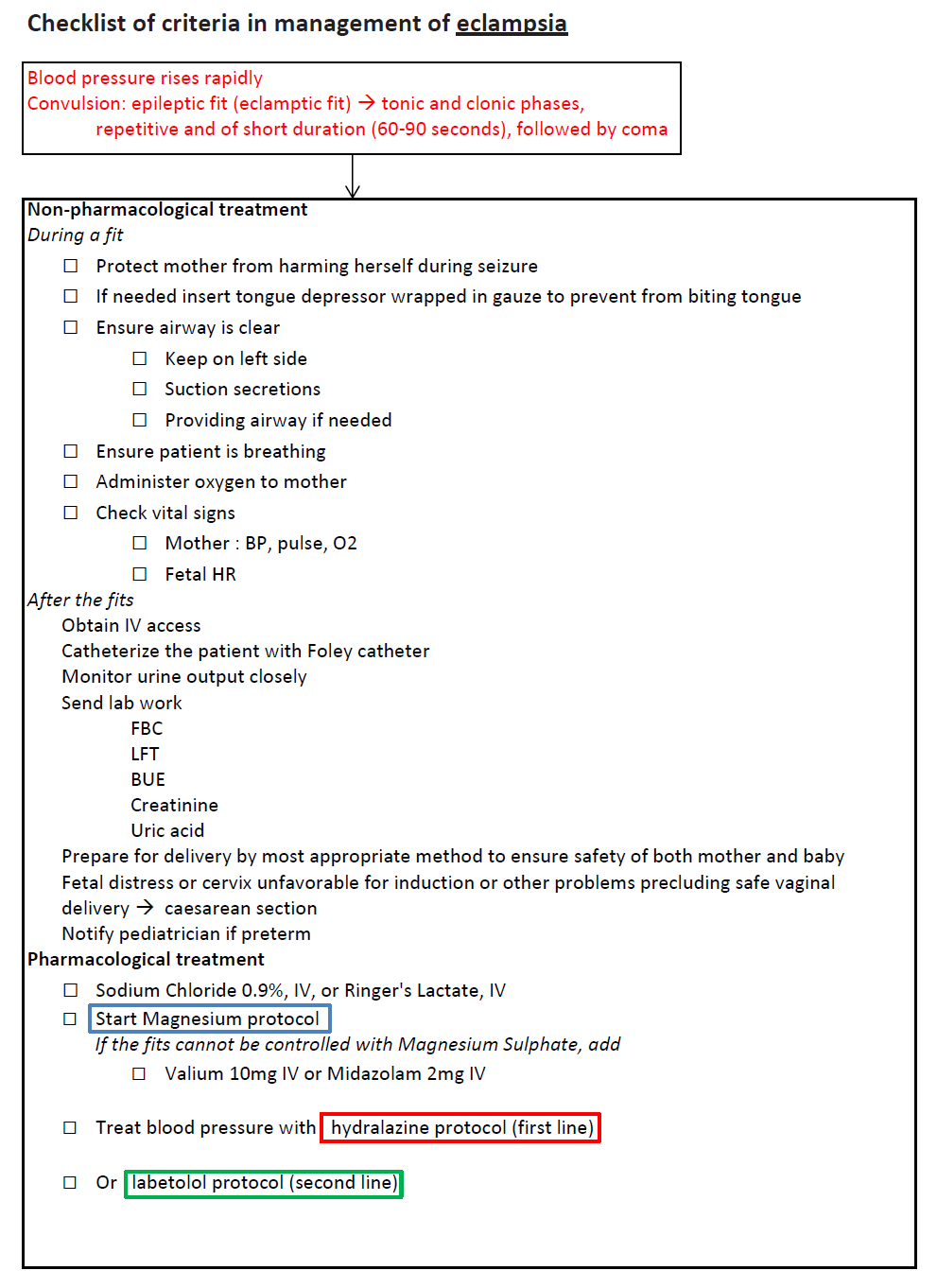

Supplement: S1 Fig — (ZIP) [file pone.0125749.s001.zip › Supplement 1-6.tif]
